# Recent Advances in Silicon Nanowire Biosensors: Synthesis Methods, Properties, and Applications

**DOI:** 10.1186/s11671-016-1618-z

**Published:** 2016-09-17

**Authors:** Pooria Namdari, Hadis Daraee, Ali Eatemadi

**Affiliations:** 1 Mechanical Engineering, Sharif University of Technology, Tehran, Iran; 2 Department of Medical Biotechnology, School of Advance Science in Medicine, Tehran University of Medical Sciences, Tehran, 69971-18544 Iran

**Keywords:** Silicon nanowires, Biosensor, Synthesis, Morphology, Biomolecule sensing

## Abstract

The application of silicon nanowire (SiNW) biosensor as a subtle, label-free, and electrical tool has been extensively demonstrated by several researchers over the past few decades. Human ability to delicately fabricate and control its chemical configuration, morphology, and arrangement either separately or in combination with other materials as lead to the development of a nanomaterial with specific and efficient electronic and catalytic properties useful in the fields of biological sciences and renewable energy. This review illuminates on the various synthetic methods of SiNW, with its optical and electrical properties that make them one of the most applicable nanomaterials in the field of biomolecule sensing, photoelectrochemical conversion, and diseases diagnostics.

## Review

### Introduction

Silicon nanowire (SiNW) biosensors are typical field effect transistor (FET)-based devices, made up of three electrodes. The mechanism of their sensing process is due to the variation in their charge density that leads to changes in the electric field at the external surface of the SiNW. Practically speaking, the resistivity of the device is increased when a negatively charged biomolecules species is synthesized with the external surface of an n-type SiNW. Furthermore, rare properties like great surface-to-volume ratio, tunable electrical and optical properties, and biocompatibility possessed by SiNW have made them good candidates for the detection of metal ions species, nucleic acids, and virus (Table 1).

**Table 1:** The performance of SiNW FET biosensors

Device specification	Fabrication	Mechanism	Application	Detection limit
p-Type SiNW, diameter: 20 nm	Bottom–up	Biotin–avidin binding	Streptavidin	10 pM
n-Type SiNW, p-type SiNW, diameter 20 nm	Bottom–up	Antibody–antigen interaction	PSA, CEA, Mucin-1	PSA 2 fM, CEA 0.55 fM
p-Type SiNW, diameter 20 nm; p-type SiNW, diameter 20 nm	Bottom–up	PNA–DNA hybridization	DNA	10 fM
p-Type SiNW, diameter 20 nm	Bottom–up	Antibody–virus interaction	Influenza A virus	Single virus
n-Type SiNW, p-type SiNW, thickness 40 nm, width 50–150 nm	Top–down	Biotin–avidin binding	Streptavidin	10 fM
n-Type SiNW, p-type SiNW; thickness 40 nm, width: 50–150 nm	Top–down	Antibody–antigen interaction	PSA, CA 15.3	PSA 2.5 ng/mL
n-Type SiNW, p-type SiNW, width 20 nm, length 30 nm	Top–down	DNA–DNA hybridization	DNA	10 pM
n-Type SiNW, p-type SiNW, width 50 nm, length 20 nm	Top–down	DNA–DNA hybridization	DNA	25 pM
n-Type SiNW, thickness ≤40 nm	Top–down	Antibody–antigen interaction	PSA	30 aM
p-Type SiNW, diameter 30–60 nm	Bottom–up	Protein–protein interaction	TnI	7 nM
n-Type SiNW, width 50 nm, thickness 60 nm, length 100 nm	Top–down	PNA–DNA hybridization	DNA	10 fM
n-Type SiNW, width 50 nm, thickness 60 nm, length 100 nm	Top–down	Antibody–antigen interaction	cTnT	Ifg/mL
n-Type SiNW, width 50 nm, thickness 60 nm, length 100 nm	Top–down	PNA–DNA hybridization	RT-PCR product of DEN-2	10 fM
n-Type SiNW, width 50 nm, thickness 60 nm, length 100 nm	Top–down	PNA–RNA hybridization	microRNA	1 fM
n-Type SiNW, width 50 nm, thickness 60 nm, length 100 nm	Top–down	Protein–DNA interaction	ER	10 fM
n-Type SiNW, width 50 nm, thickness 60 nm, length 100 nm	Top–down	Antibody–antigen interaction	cTnT, CK-MM, CK-MB	1 pg/mL

The three electrodes making up a SiNW consist of a source and drain an electrode that connects the semiconductor channel together and the third electrode; gate electrode regulates and maintains the conductance of the channel. It should be noted that the ability of this device to sense is as a result of the location of SiNW between the source electrode and the drain electrode in the semiconductor channel (Table 2).

**Table 2:** Brief comparison of different SiNWs alignment methods

Alignment type	Alignment method	Inter-NW distance, alignment yield and control of NW density	Merits	Demerits	References
Langmuir–Blodgett alignment	Parallel alignment of SiNWs during uniaxial compression of Langmuir–Blodgett trough	8–10 NW/mm; alignment yield is about 80–90 %. SiNW density is controlled by the compression of Langmuir–Blodgett trough	Alignment can be useful has a substrates spanning several cm^2^ in area. Cross-sINW structure is attainable using sequential rounds of Langmuir–Blodgett alignment.	Irreproducibility in the alignment direction of sINWs can lead to bad/weak end-to-end registration with the source and drain electrodes. It is only effective with SiNWs with diameter >15 nm. Almost impossible to control and coordinate the number of SiNWs bridging the source and drain contact electrodes	[17]
Blown–bubble alignment	Suspension of SiNW–polymer solution blown into a bubble using gas flow	ca. 1 NW/3 mm Alignment yield: 90 %. SiNW density is organized by varying the concentration of SiNWs in the SiNW–polymer suspension solution.	Alignment method can be applied to various SiNW materials like planar, plastic, curved. Alignment feasible up to various length scales (from mm to m).	Needs surface functionalization of SiNWs with epoxy group to form SiNW-polymer film, which may reduce the availability and efficiency of SiNW surface in terms of immobilization of biorecognition element Hard to control the number of SiNWs bridging the source and drain contact electrodes	[18]
Flow-based alignment	Microfluidic flow-driven shear forces, where the adsorption of NWs is facilitated by surface charge.	2–3 NWs/mm Alignment yield: 80 %. SiNW density is controlled by flow duration.	Cross-SINW arrays and equilateral triangles can be constructed using a chemically patterned surface and sequential layer-by-layer assembly steps with different flow directions. Alignment needs small sample volume of SiNWs (mL).	Alignment is restricted to planar substrates and to small length scales ranging from few mm to cm. It is only applicable to SiNWs with diameter >15 nm. It is so difficult to control the number of SiNWs bridging the source and drain contact electrodes.	[19]
Electric-field based alignment	It involves balance of hydrodynamic and dielectrophoretic forces.	1 NW/12 mm Alignment yield: >98 %. NW density is controlled by the number of patterned electrode sites in a specific area.	There are no available incorporation issues of SiNWs with the source and drain contact electrodes. Surface modification of SiNWs can be done before alignment. Each SiNW can be worked on singly from an electrical contact standpoint.	It demands precise control of the hydrodynamic and dielectrophoretic forces. Dissimilarities in the physiochemical properties of SiNWs can truncate the alignment process. Alignment only possible for small area (from mm^2^ to cm^2^). The quality and density of the SiNW produced is low as compared to other methods.	[20]
Contact printing alignment	Shear stress during the sliding of donor (the growth substrate) and receiver substrates. An intermediate step such as stamp transfer using a roller can also be employed (roll-transfer printing).	4–8 NW/mm Alignment yield: 80–90 % NW density can be controlled by changing the receiver substrate with various functional groups.	Alignment viable with several SiNW materials and can be applied to diverse substrates (silicon, plastic and rubber etc.). Also applicable to SiNWs with diameter <15 nm Multilayer functional device structures are achievable by iterative contact printing and device fabrication steps. Roll-transfer printing method can be operated in a continuous fashion. Strained PDMS stamp can be applied to improve the efficiency of alignment yield and SiNW density.	Lack of control in breakage of SiNWs during the transfer process, resulting in distribution of NW lengths. The length of SiNWs printed on the receiver substrate is characteristically less than the length of SiNWs on the growth substrate. It is difficult to control the number of SiNWs bridging the source and drain contact electrodes.	[21, 22, 24]

### SiNWs Synthesis Techniques

Generally, there are presently two procedures that have been developed for the nanofabrication processes of SiNWs, and they include top–down approach (Fig. 4) and bottom–up approach (Fig. 5). The efficient performance of the SiNW biosensor can be determined by various factors like diameters, carrier densities, and surface chemistry. An in-depth discussion about the bottom–up of the synthesis of SiNWs has been reported by Ramanujam et al. [1]. The bottom–up approach includes processes like vapor-liquid-solid (VLS) and oxide-assisted growth (OAG) and photolithography or e-beam lithography [2]. VLS technique has been reported to adopt to synthesize SiNWs, along with their applications as biosensors. The bottom–up method involves the synthesis of the SiNWs from a mass of silicon wafer with the reaction been metal catalyzed, while top–down technique begins from a bulk silicon wafer and trims down to the preferred and required size and shape of SiNWs through a lithographic mechanism. For comparison, see Tables 3 and 4.

**Table 3:** Showing SiNWs synthesis techniques

Techniques	Types	Material	References
Bottom–up approach (Fig. 5)	Vapor-liquid-solid (VLS) Oxide assisted growth (OAG) Metal-assisted chemical etching	Coating-catalyzed metals on silicon substrate (CVD) Coating-catalyzed metals on silicon substrate-laser ablation Si wafer-coated metal catalyst introduced with Si gas source OAG-thermal evaporation OAG-HF Electroless metal deposition-chemical etching	[4] [5, 6] [27] [11, 13, 15]
Top–down approach (Fig. 4)	None	Electron beam lithography Nanoimprint lithography DEA technology and photolithography Photolithography-DRIE-TMAH-thermal oxidation Angled thin-film deposition-micrometer scale photolithography Lateral bridging growth	[28] [29] [30] [31] [32]

**Table 4:** Differences between top–down and bottom–up approach synthesis of SiNWs

	Top–down approach	Bottom–up approach
Device preparation	SiNW and device development were done by etching a silicon-on-insulator (SOI) wafer.	SiNWs are produced from molecular precursors by using a metal nano-cluster mediated VLS mechanism.
	Fabrication techniques are developed from technology like optical lithography, reactive ion etching, e-beam lithography, and anisotropic wet etching	For a transmission electron microscope (TEM) image, check Fig. 5b.
	For a scanning electron microscope (SEM) image, check Fig. 5c.	
Merits	Docile to mass production	Easiness in the choice of material for nanowire development
		Alignment and directional control of the growth of nanowire crystal is possible [33].
	Dependability and reproducibility of the synthesizing process	Various doping levels and high availability of dopants can be introduced during the synthesis.
	No integration problems	There is high possibility of synthesizing SiNWs of diameter less than 10 nm.
	SiNWs with several cross sections like triangular [34] and trapezoidal can be fabricated as its essential for selective functionalization of SiNWs [35].	Appropriate for fabricating multilayer SiNW device structures
	SiNWs with double-gate structures that are reinforced on a co-planar geometry can be produced (improved sensitivity) [36, 37].	Flexible to incorporation with flexible and transparent device substrates [38]
Demerits	Demands a lot of time for processing	Leads to distribution of lengths and measurements of the synthesized SiNWs
	Costly	Device development involves precise arrangement and positioning of SiNWs resulting to integration problems.
	Restricted choice of materials for SiNW fabrication	Extremely hard to realize accurate control of number of SiNWs bridging the source and drain electrodes resulting to disparities in batch-to-batch fabrication of SiNW devices
	Incompatibility of surface chemistry with the tough processing of nanofabrication	Alignment problems related with long SiNWs
	Measurements of SiNWs restricted by the resolution of the fabrication process	Mass production of SiNW devices almost impossible

#### VLS

**Fig. 1:**
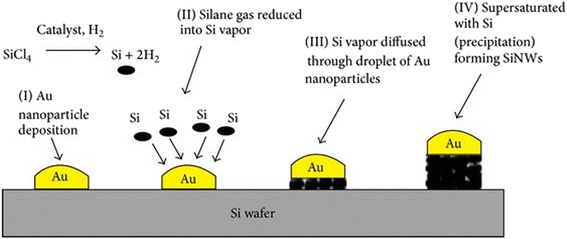
The silicon nanowire biosensor synthesis using VLS method via CVD method: Step (i) Gold nanoparticle deposition. Step (ii) Reduction of silane gas to silicon vapor. Step (iii) Diffusion of silicon vapor via gold nanoparticles. Step (iv) Formation of SiNWs via super-saturation with silicon. This figure was reproduced from [7]

Silicon nanowire synthesis via VLS was first reported in 1964 using silicon substrate integrated with liquid Au droplet. In VLS, there is a deposition of metal-catalyzed (Au, Fe, Pt, Al, etc.) on the silicon wafer and then the SiNWs growth is augmented either by chemical vapor deposition (CVD) technique [3, 4] (Fig. 1). Essentially, silicon wafer coated with metal catalysts are positioned at the middle of a tube furnace and initiated with a silane (SiH4) or tetrachlorosilane (SiCl4) and passed above the metal catalyst accumulated on Si wafer in the chamber at above eutectic temperature [5].

The SiH4 gas serving as the source of silicon gas would be converted into silicon vapor and disperses through a metal catalyst to produce metal-silicon alloy droplets. As silicon diffuses across the metal nanoparticle catalyst leading to a supersaturated state of condition, the silicon will precipitate out from droplets of metal-Si forming silicon nanowires [6].

#### OAG via Thermal Evaporation

**Fig. 2:**
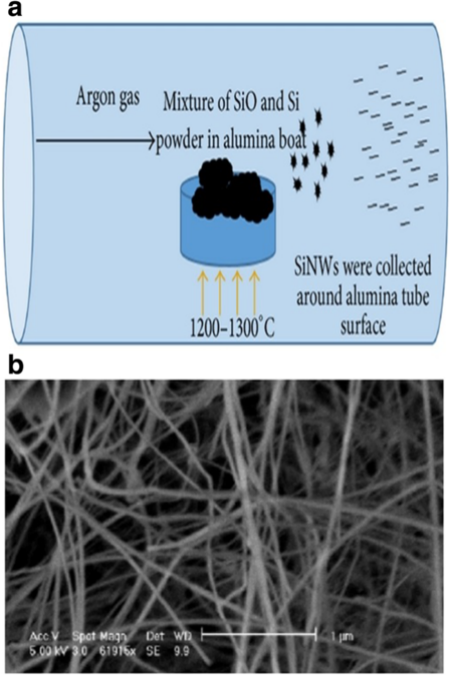
**a** Illustrated diagram showing synthesis of SiNWs via OAG method. **b** SEM image of synthesis of SiNWs via OAG method. These figures were reproduced from [9]

Recently, many researchers have effectively synthesized SiNWs via a bottom–up approach called OAG via thermal evaporation due to its in generating a huge quantity of SiNWs [8]. Using OAG method, the growth of SiNWs was significantly improved using SiO as starting material to stimulate the nucleation and the growth of SiNWs without the use of catalyzed metal generating high purities SiNWs and free of metal impurities [9]. The development of SiNWs using OAG method has been reported by Shao et al. [9]. Briefly, they reported that the alumina boat holding the mixture of SiO powder (10 g) and Si powder (0.05 g) was positioned at the alumina tube, inside a tube furnace. At particular pressure, Argon was introduced as a carrier gas and for 10 h, the furnace was heated to a temperature of 1250–1300 °C. The resulting SiNWs are with a diameter of 85 nm and were gathered around the alumina tube surface (Fig. 2). One of the features of the produced SiNWs via OAG method is it possesses at its outer layer, an oxide layer that is chemically inert. To efficiently improve the electrical and optical properties of the produced SiNWs, the outer layer covered by oxide layer should be removed by treating the oxide layer with hydrofluoric acid (HF).

It should be noted that this method is more preferable to VLS as it enables to produce SiNWs with various morphologies in chains, rods, wires, ribbons, and coaxial structures, and the use of silicon sources like silane (SiH4) or SiCl4 can be circumvented.

#### Metal-Assisted Chemical Etching

This is the most low-cost and simple method of synthesizing SiNMs [10]. This method comprises two main stages which are electroless metal (silver, nickel, platinum, gold) deposition on silicon wafer followed by chemical etching in fluoride-ion-based solution [11, 12]. The real-time reaction of electro-less deposition and chemical etching has been reported by Brahiti and co-workers [13], and it entails soaking of cleaned silicon wafer into NH4HF2 and AgNO3 solution.

**Fig. 3:**
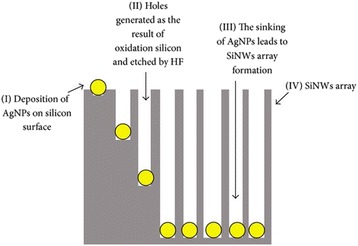
Silver-assisted chemical etching mechanism: Step (i) Deposition of silver nanoparticles on silicon surface. Step (ii) Generation of holes via the oxidation of silicon and etching by HF. Step (iii) Formation of SiNWs arrays leads to silver nanoparticle sinking. Step (iv) Newly formed SiNWs. The figure was reproduced from [15]

In this method, silver ion attracts electrons from the silicon substrate that stemmed from the deposition of silver nanoparticle on silicon surface [14]. The silicon underneath the silver nanoparticle is oxidized and holes are formed by the action of HF; the holes formed serve as a sinking route for the residual of the Ag nanoparticles thereby forming a longitudinal and lateral suspension of silicon generating the formation of SiNWs arrangements [15] (Fig. 3). Zhang and co-workers [16] also reported that when parameters like temperature, concentration and deposition time, and doping level are manipulated, diverse morphologies of SiNWs arrays could be produced.

#### Alignment OF SiNWs

The bottom–up approach for the synthesis of SiNWs is appealing as it leads to the production of efficient high-quality, minute diameter of about 3–5 nm and single-crystalline SiNWs. However, it should be noted that in order to utilize SiNWs in the fabrication of any device, SiNWs need assemblage with the coordinated transfer, alignment, and density on a device substrate for successive incorporation with the circuitry in a spatially defined approach.

Various methods have been reported in several kinds of literature that permit well-ordered configuration and assembly of one-dimensional SiNWs into a required design. These methods include Langmuir–Blodgett (LB), blown–bubble (BB), microfluidic flow, electric field, and contact printing alignment. In brief, the details of these methods are described in Table 2 below.

#### Top-Down Approach

**Fig. 4:**
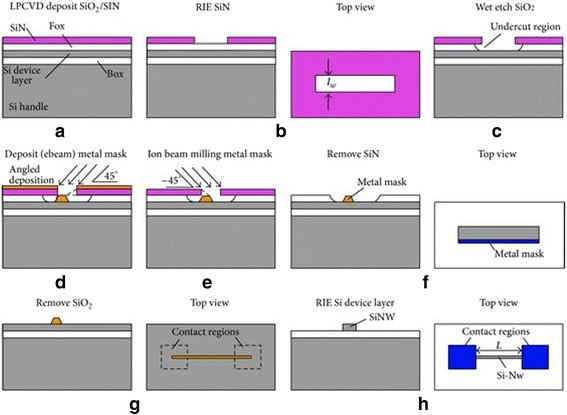
Silicon nanowires via DEA fabrication process. Step (**a**) deposition of SiO_2_ by LPCVD. Step (**b**) reactive ion etching (RIE) in the Si3N4 layer. Step (**c**) Undercut the wet etch SiO_2_. Step (**d**) deposit the metal mask around the undercut region. Step (**e**) ion bean milling the metal mask. Step (**f**) hard-etch metal mask layer. Step (**g**) Remove the silicon nanoparticle. Step (**h**) Remove SiO_2_ Step (vii) the newly formed SiNW. This figure was reproduced from [30]

Presently, there are two approaches to fabricate SiNW devices, namely top–down and bottom–up [2]. Several researchers have reported in details the modalities behind top–down approaches for fabrication of SiNW [23, 26]. Table 2 below shows some differences between the two fabrication approaches as regards the merit and demerit related with each fabrication techniques. The top–down approach involves the synthesis of SiNWs starting from the bulk material and scaled down into a distinct SiNW that can be produced via the process of nanolithography techniques like electron beam lithography (EBL) [28] and nanoimprint lithography and so on [29]. The synthesis via top–down technique has been reported by Park and co-workers [28] by using electron beam lithography and reactive ion etching on silicon-on-insulator (SOI) wafer leading to the production of high-pitched control of the geometry and alignment of SiNWs with efficient electrical properties. In addition, Vu and colleagues demonstrated SiNW arrangement with width dimensions of 20-nm width and 60-nm height [29] which possess the features of both nanoimprint lithography and wet anisotropic etching. Furthermore, the use of DEA technology and photolithography technique has been reported by a group of researchers to produce a lone SiNW with radius below 50 nm and 1 mm in height [63] (Fig. 4).

**Fig. 5:**
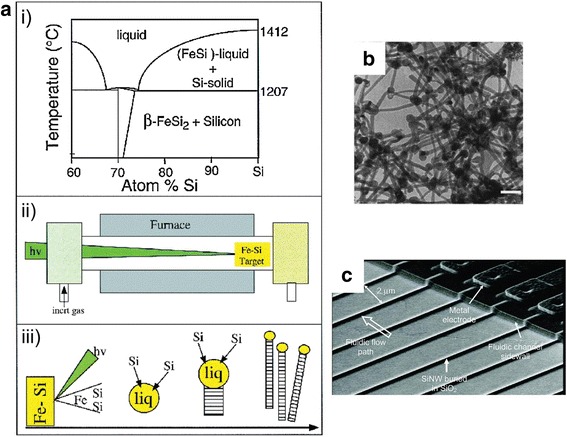
**a** Bottom–up approach synthesis of SiNWs. (i) Phase diagram for Fe–Si binary system. (ii) Schematic diagram showing synthesis of SiNWs via VLS growth and laser ablation cluster formation method. (iii) Growth profile for the synthesis of SiNWs. **b** TEM image of SiNWs synthesized through bottom–up approach using VLS techniques. **c** SEM image of SiNWs method via top–down approach. These figures were reproduced from [39] and [40]

In another recent work by Kulkarni and co-workers, they were able to efficiently fabricate SiNW arrays of about 250 nanowires with dimensions of 150 nm breadth and 20 μm in length with 3.2 nm similar space size via top–down approach [31]. It should be noted that they adopted the four stages of photolithography techniques in their research (Fig. 5).

### Properties of Silicon Nanowires

#### Electronic Properties

The electronic and electrical properties of SiNW greatly and strongly depends on growth direction, size, morphology, and surface reconstruction because of their small sizes which are so evident in the size dependence of the electronic band gap width of SiNWs regardless of wire direction. The diameter of the wire is inversely proportional to the width of the band gap resulting into a deviation from the bulk silicon. In addition, the alignment of the wire axis and its surface area has some effects on the electronic properties of SiNWs.

**Fig. 6:**
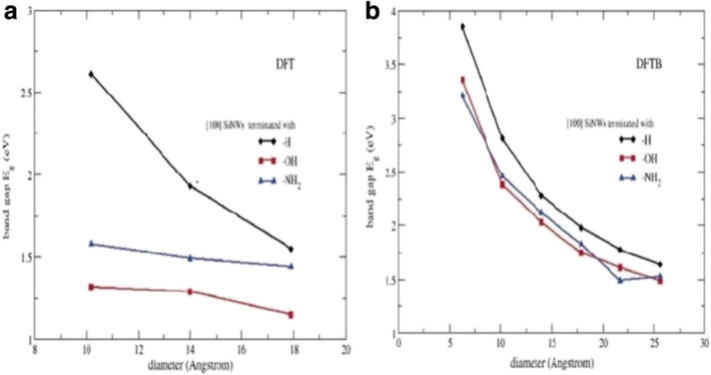
Showing band gap as a function of the silicon nanowire diameter for various surface terminations. **a** DFT calculations within GGA-PBE. **b** Results from a density-functional tight-binding (DFTB) parameterization. This figure was reproduced from [41]

Michael Nolan and co-workers investigated the band gap modification for small diameter of about 0.9–1 nm silicon nanowires fabricated by the use of several types of surface termination by density functional theory calculations (Fig. 6). The 0.9–1-mm nanowire demonstrated a direct band gap that increases concomitantly with a decrease in the diameter of the wire because of quantum limitation, regardless of surface termination.

Furthermore, Sacconi and co-worker also demonstrated the electronic properties of silicon nanowires with varying approaches such as Empirical Tight-Binding (ETB) model, the Linear Combination of Bulk Bands (LCBB) model, and Non-Equilibrium Green Function (NEGF) model by investigating both hydrogenated and SiO_2_ terminated silicon surfaces in these models.

The diameter of SiNW reduced from 3.2 to 1.6 nm concomitantly with an increase in the band gap of hydrogenated nanowire from 1.56 to 2.44 eV. However, Sacconi reported a minute increase in the SiO_2_/SiNW structure. This phenomenal is as a result of lower restriction caused by SiO_2_ trapping the SiNW when compared to simple hydrogen termination. They also reported effective masses for conduction and valence bands. Reduction of the conduction mass, from 0.47 m_o_ to 0.31 m is equal to the effect of increasing the thickness of silicon on a hydrogen-terminated wire but the effect on the SiO_2_-confined wire was the same as a result of increase in silicon thickness and a decrease in effective mass from 0.36 to 0.29 m_o_ [42].

#### Optical Properties

Silicon bulk possesses an indirect band gap coupled with the valence band maximum at the Γ point and the conduction minimum at about 85 % along the Γ to X direction, and a phonon is needed to sustain the momentum in any electronic transition. Outstandingly, SiNWs developed laterally and most of the crystallographic orientations have a direct band gap; as a result, both the maximum and minimum of the valence band and the conduction band respectively occur at a similar point in k-space. This unique property has made SiNWs as effective optically active materials for photonics applications. Controlling the band gap width can open new doors to the application of SiNWs in optoelectronics fields: such that both the band gap and width of SiNWs can be tuned to increase its optical efficiency. The possibility of tuning the band gap and width of SiNWs is determined by controlling the chemical composition and the coverage density of the wire surface area, and it has been regarded as an easier and effective route for tuning. Leu and co-workers reported that chlorine, bromine, and iodine can be used in place of hydrogen as a surface passivation agents because they have the ability to reduce the band gap but still maintaining the semiconducting abilities of the wires [43].

Recently, Ramos and colleagues demonstrated the optical and mechanical characterization of SiNWs by showing experimental and theoretical data to investigate the fundamental mechanisms behind the light-nanowire interaction in an optical interferometry setup [44].

**Fig. 7:**
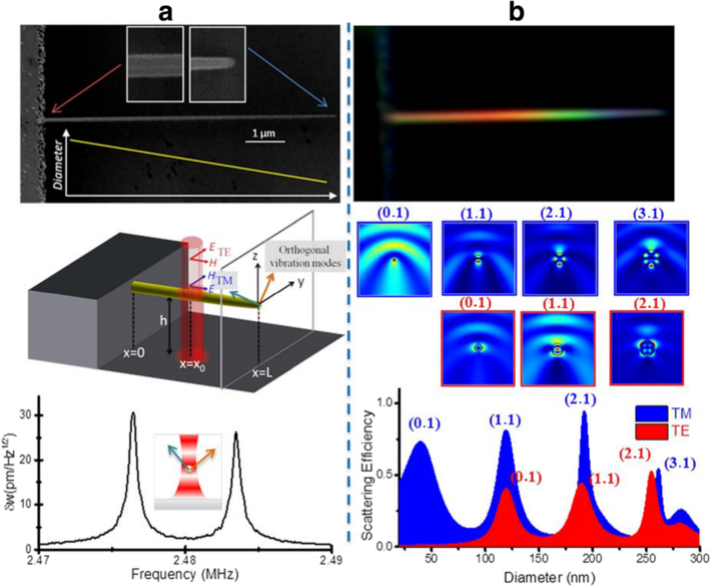
**a** Scanning electron microscopy of a tapered nanowire. **b** Optical dark field image of the nanowire and theoretical calculation of the scattering efficiency of the nanowire for a wavelength of 633 nm as a function of the diameter. This figure was reproduced from [45]

In the experiment, they synthesized silicon nanowires horizontally compiled and epitaxially clamped at the sidewalls of pre-patterned micro-trenches on Si substrates via the vapor-liquid-solid approach [45]. The length and diameter of the fabricated nanowires were between 8 and 16 μm and 40 and 240 nm, respectively, and a vertical distance between 1.0 and 1.3 μm was between the nanowire and the substrate underneath. One of the most important features of this study was the selection of growth conditions to synthesize tapered nanowires in which the diameter linearly decreases from the clamped end to the free end [46]. Optical interferometer at room temperature was used to measure the mechanical vibration of the nanowires [47] in a Fabry-Perot configuration operating at a wavelength of 633 nm. Figure 7a depicts a scanning electron microscopy (SEM) diagram of one of the fabricated tapered nanowires and a frequency spectrum of the thermo-mechanical oscillations that displays the quasi-degeneration of the two orthogonal fundamental vibration modes [47]. The fabricated tapered nanowire has a length of 11.3 μm, and there was a reduction in the diameter from 150 ± 5 nm at the clamp area to 60 ± 5 nm at the tip as determined by the electron microscopy.

The tapered nanowire displayed colors ranging throughout the visible spectrum between the clamped and loose ends when it is observed under a dark-field microscopy (Fig. 7b) [48, 49]. Given that the dark-field microscopy has the optimum capability to detect only scattered light, as such the emitted colors of the collected light solely comes from the light scattered by the tapered nanowire. In addition, Ramos et al. reported that numerical simulations from their studies of light-silicon nanowire interaction demonstrated that silicon nanowires display optical resonances that competently improve the light scattering for a specific wavelength values to diameter ratio. These optical resonances created a connection between the diameter of the tapered nanowire and the scattered light’s color similar to that of the dark-field data [45].

Ramos et al. in order to investigate more on the optical resonances of the tapered nanowire, at a wavelength of 633 nm, the scattering efficiency of the nanowire was calculated as a function of its diameter that is depicted in Fig. 7b for transverse magnetic (TM) and transverse electric (TE) azimuthal polarizations [45]. The spectra illustrated a series of optical resonances indicating the location of strong scattering of light while the light limitation within the nanowire is been demonstrated by the spatial distribution of the near electric-field intensity at these resonances (Fig. 7b). It should be noted that there is a generation of evanescent field resulting from the electromagnetic field extending some few nanometers away from the nanowire because of the small size of the nanowire and thus the resonances can proficiently relate with the neighboring electromagnetic field [50].

### Application of SiNWs

#### SiNWs as Ion-Selective Nanosensors

**Fig. 8:**
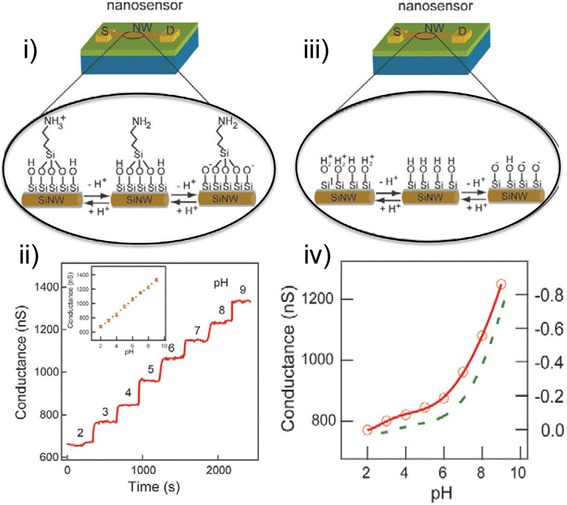
SiNW sensor for pH measurements. (**i**) Graphics representation of an APTES modified SiNW. (**ii**) Real-time modifications in conductance response of APTES-modified p-type SiNW. (**iii**) Graphics representation of an unmodified SiNW. (**iv**) Conductance response of an unmodified SiNW as a function of pH. This figure was reproduced from [51]

Cui and co-workers reported the first case of SiNW application as chemical transducers in 2001 [51] (Tables 5 and 6). Cui and colleagues produced a pH nanosensor by modifying p-type (boron-doped) SiNWs with an APTES film. Depending on the pH of the solution, the APTES film was subjected to the process of protonation or de-protonation, which regulated the surface charge on the SiNWs and gated the conductance of the NWs in a pH-dependent manner (Fig. 8ii).

**Table 5:** Showing selected applications of DNA-based SiNW-FET sensors

Capture probe (target length)	Limit of detection (LOD)	Buffer composition, ionic strength, Debye screening length	Description	Reference
DNA (16 BP)	10 pM	1 SSC, 165 mM, ca. 1 nm	Electrostatically adsorbed capture probe, oxide layer removed by etching	[59]
PNA (22 BP)	10 fM	0.01 SSC, 1.65 mM, 7.0 nm	Oxide layer removed by chemical etching and SiNW surface passivated with an organic film	[60]
PNA (22 BP)	1 fM	0.01 SSC, 1.65 mM, 7.0 mm	Electrostatically neutral analog of DNA as a capture probe	[55]
DNA (19 BP)	1 fM	0.1 PBS, 15 mM, 2.3 nm	Small size of SiNWs achieved by implementation of NW structures with triangular cross section	[61]
DNA (24 BP)	0.1 fM	0.01 PBS, 1.5 mM, 7.3 nm	Triangularly shaped SiNW-FETs operated at “subthreshold” regime	[34]
DNA (15 BP)	0.1 fM	0.01 PBS, 1.5 mM, 7.3 nm	Alignment of interfacial chemistry by electric field	[54]
DNA (30 BP)	50 aM	0.1 PBS, 15 mM, 2.3 nm	RCA amplification	[62]

**Table 6:** Showing application of SiNWs in sensor technologies

Methods	Application	Reference
Surface-enhanced Raman scattering	Amoxicillin, calcium dipicolinate, protein, immunoglobulin	[68]
Fluorescence sensor	Multiplex DNA detection	[57]
	Protein immunosensor	[65]
		[69]
	NO detection	[70]
	Ln (III) detection	
Electrochemical sensor	H_2_O_2_ detection	[71]
	Dopamine	[72]
	Glutathione	
	BSA	[73]
Field effect transistors	DNA detection and hybridization	[34, 56]
	CRP and PSA detection	[74]
	Lectin EC detection	[67]
	Interleukin-I genes	[75]
	Influenza virus	[76]

It can be seen that the SiNW conductance increased concomitantly with an increase in pH from 2 to 9 in a linear manner, and at a specific pH value, the conductance of the SiNW was constant. Figure 11a(i) shows the surface modification of SiNW with APTES with an introduction of primary amine functional groups to the underlying surface silanol groups that resulted into the protonation of NH_2_ group into NH_3_^+^ at low pH (Fig. 8i). The density of the charge carriers in the p-type SiNW was depleted as a result of the positive surface charge thereby leading to a reduction in conductance. At an alkaline pH, the surface silanol groups were deprotonated to SiO (Fig. 8iii), leading to a concomitant accumulation of charge carriers in the p-type SiNW and increase in conductance. However, conductance measurements carried on unmodified SiNWs demonstrated a non-linear dependence on changes of pH (Fig. 8iv).

Recently, Chen and co-workers also reported that the functional group present on the surface of a SiNW was responsible for the pH sensitivity of the SiNW-FET sensor [52]. Furthermore, it has also been reported that Dorvel et al. produced SiNW-FETs via top–down method with hafnium oxide (HfO_2_)-based gate dielectric interfaces for pH sensing that gave a response of ca. 56 mV/pH [25]. However, there is a possibility that the pH sensitivity of NW-FET sensors can exceed the Nernst limit by operating the device under dual gate [53] or in a DG configuration [36].

#### Nucleic Acid and DNA Detection Using SiNWs

**Fig. 9:**
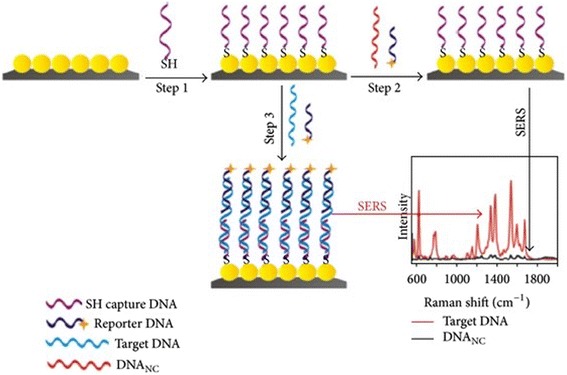
Illustrated diagram of surface enhanced Raman scattering (SERS) sensor-based SiNWs/AgNPs for DNA detection. Step (i) DNA capture by SH. Step (ii) Addition of reporter DNA. Step (iii) Targeting DNA. Step (iv) Graphical illustration of target DNA and DNANC. This figure was reproduced from [57]

Nucleic acids have been reported to be labeled and successfully detected by SiNW-FETs thereby making them attractive sensors. The negative charge related to the sugar-phosphate backbone of DNA and RNA allows sensitive detection of nucleic acids with detection limits in the fM range [34, 54] (Table 5). DNA probes that are electrostatically neutral can be used to attain comparative changes in surface charge and this is evident in the use of PNA [55] and alkyl-phosphonate oligonucleotide [56] chemistries in probe production that lead to an enhanced signal-to-noise ratio as compared to DNA. In addition, Jiang and co-workers [57] have produced a SiNW integrated with AgNPs through metal-assisted chemical etching method-based sandwich structural DNA SERS sensor for multiplex DNA detection. Jiang and colleagues reported that immobilization of thiolated single-stranded DNA probe functionalized with silver nanoparticles through Ag-S bonding and hybridization with the target reporter probe marked with Rhodamine 6G before SERS detection was done (Fig. 9). This significant approach demonstrated high reproducibility and specifically for DNA detection coupled with the fact that SERS sensor is efficient of distinguishing single base mismatched DNA at lower concentrations of 1 pM.

**Fig. 10:**
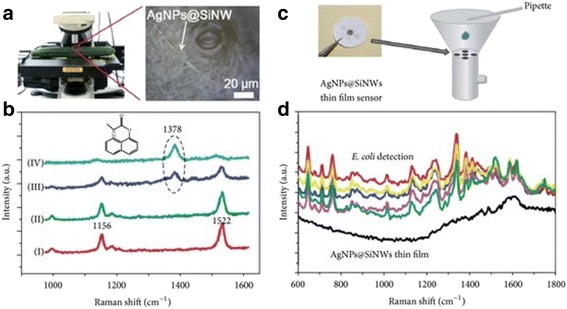
**a** Showing photograph of the detection of pesticide deposits on a cucumber surface experiment. **b** Showing Raman spectra documented from the rough cucumber surface with 1-s gain time and ×50 objective. **c** Showing photograph of SiNWs compiled on a commercially available filter film and graphic picture of the *E. coli* detection. **d** Showing Raman spectra documented from a blank thin film and five different sites on the *E. coli*-contaminated AgNP@SiNWs thin film with 10-s acquisition time and ×50 objective. This figure was reproduced from [58]

Han et al. [58] reported the optimized single SiNWs-AgNPs for surface-enhanced Raman scattering detection of pesticide residues (carbaryl) on the surface of a cucumber in regard to merits like a rapid response, easiness, elasticity, and increased resolution. Han and colleagues also demonstrated the discovery of *Escherichia coli*-based SERS sensor by filtering the AgNPs-SiNWs because the water has been contaminated with *E. coli* and then followed by characterization by Raman spectroscopy (Fig. 10a, b).

#### Fluorescence’s Sensor-Utilized SiNWs

**Fig. 11:**
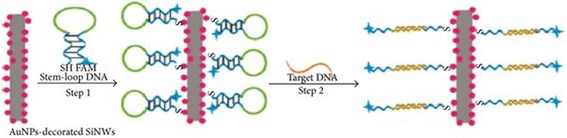
Illustrated diagram showing preparation of silicon-based nano-MBs for DNA analysis. Step (i) Fabricating AuNP-decorated with SiNWs with SH-FAM stem loop DNA. Step (ii) Reaction with target DNA. This figure was produced from [63]

**Fig. 12:**
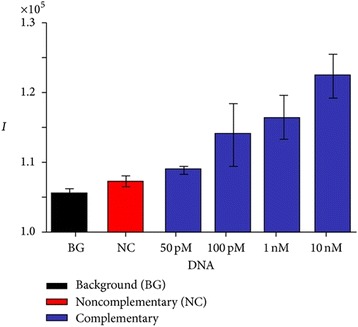
Illustrated graph showing fluorescence intensity of various concentrations of complementary target DNA with the complementary bar at 10 nM showing the highest fluorescence intensity and the background bar showing the lowest fluorescence intensity. This figure was produced from [65]

Recently, Su and co-workers [63] demonstrated a novel AuNP-SiNW-based molecular beacons (MBs) for high-sensitivity multiplex DNA detection (Figs. 11 and 12). They reported that AuNP-SiNW-based MBs displayed stout stability in wide salt concentrations within the range of 0.01–0.1 M and thermal stability within 10–80 °C. And in addition, it slowly accumulated as a result of the salt-induced reduction of electrostatic between AuNPs at an increased concentration of salt [64]. Su and co-workers reported that after the process of DNA hybridization, there were conformational changes in the stem loop of MBs leading to spatial separation of the carboxyfluorescein and AuNPs-SiNWs, thus improving the fluorescence intensity.

Finally, Su discovered that the fluorescence intensity was significantly augmented with an increased concentration of target DNA from 50 pM to 10 nM, Conclusively, the authors reported that AuNPs-SiNWs based on MBs were efficient in detecting DNA target at reduced concentrations down to pM level and also exhibited high selectivity in the presence of non-complementary DNA and single base mismatch.

Furthermore, Han recently demonstrated another application of SiNWs [65] for fluorescence protein immunosensor development. They reported the construction of vertically aligned SiNW arrays with a dimension of 8 μm in height and 75 μM in radius through electro-less etching (AEE) process, and protein were covalently trapped onto APTES-modified SiNWs.

**Fig. 13:**
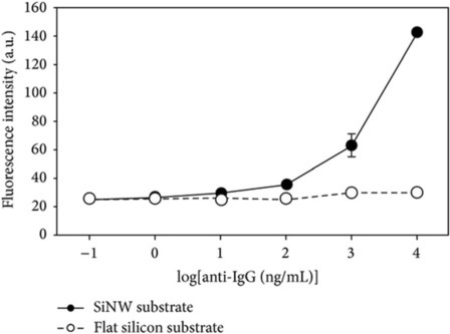
Schematic graph showing change in fluorescence intensity with concentration of FITC-anti IgG. These figures were reproduced from [34]

**Fig. 14:**
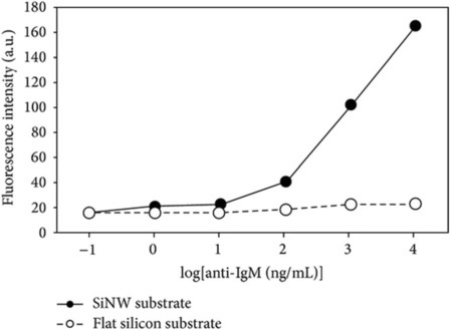
Schematic graph showing change in fluorescence intensity with concentration of Cy3-anti IgM. These figures were reproduced from [67]

As a result of high aspect ratio of SiNW-produced high surface of SiNWs that increased the immobilization of loaded BSA protein, based on this potent positive result of BSA immobilization using modified SiNWs-BSA, Han and colleagues were impressed to fabricate two types of immunosensor assays between IgG and FITC-anti-Ig-G (fluorescein isocyanate) and IgM and Cys3-anti IgM. In conclusion, they reported in their findings that fluorescence intensity due to the bond between anti-IgG and anti-IgM was greatly enhanced using SiNWs compared with planar substrates (Figs. 13 and 14).

#### FET Sensor-Utilized SiNWs

**Fig. 15:**
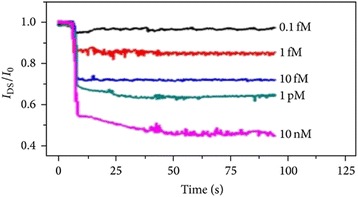
Showing a schematic plots of normalized current change against time with target DNA at various concentrations for probe DNA modified SiNW device. This figure was reproduced from [34]

**Fig. 16:**
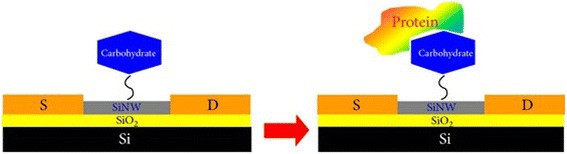
Showing schematic diagram of the SiNW biosensor for free detection of carbohydrate-protein interaction. This figure was reproduced from [67]

SiNW sensors are classic FET-based devices, composed of three electrodes. The variation in charge density can shed more light on the mechanism of the sensing process, which stimulates a change in the electric field at the SiNW outer surface. In practical, a negatively charged biomolecules species integrated to the outer surface of an n-type SiNW increases the resistivity of the device and vice versa if using p-type SiNWs [66]. More recently, Gao et al. [34] have fabricated a high performance of label-free and direct time for DNA detection using SiNWs-FET sensor via top–down approach. In their research work, they efficiently improved the sensitivity of the SiNWs-FET sensor by optimization of qualities like gate voltage, probe concentration, and buffer ionic strength. In brief, SiNW surface was firstly customized by the amine group of APTES and functionalized with carboxyl (COOH–) group modified target DNA via *N*-hydroxysuccinimide (NHS) and 1-ethyl 3-(3-dimethylaminopropyl) carbodiimide (EDC). Conclusively, Gao and co-workers reported that the enhanced SiNWs-FET sensor demonstrated a detection limit of 0.1 fM for DNA target (Fig. 15). In addition, the existing change presented around 40 % when DNA probe hybridized with full complementary target DNA and presented with only 20 and 5 % upon the introduction of single and second base mismatched DNA. It should also be noted that Zhang et al. [67] have investigated for the very first time the development of SiNWs-FET sensor based on the carbohydrate-protein interaction where unmodified carbohydrate is immobilized through the formation of an oxime bonding. Zhang and colleagues’ investigations on the newly fabricated sensor demonstrated increased specificity of lectin EC detection via galactose-modified SiNW sensor which is able to detect as low as 100 fg/m, as against 400 fg/m of other previously investigated sensors (Fig. 16).

## Conclusions

Several research works have investigated the efficiency of SiNWs and SiNW coupled with metal nanoparticles like gold and silver nanoparticles and have labeled it as excellent sensing material electrodes with high-quality catalytic activity and conductivity that can be harnessed in different fields due to their unique characterization (high detection, portability, and easiness of the procedure [77–79]. However, there are still few restraints to overcome [80–82].

Firstly, the two main broad fabrication techniques of SiNWs must be more efficiently developed to guarantee the dependable electrochemical and electrical SiNW sensor [83]. In addition, parameter manipulations in SiNW synthesis in terms of its alignment, surface area, and diameters are to be done in other to fabricate a highly controlled and reproducible sensor-based SiNWs [84].

Secondly, via the bottom–up techniques, the produced SiNW lacks control, accurate alignment, and identical precise direction as such new and improved synthesis technique is needed with greater control and accurate alignment [85–87]. Although this problem has been solved in top–down approach but its expensive cost of SiNW fabrication sensors still poses a problem for several manufacturers. Conclusively, SiNW is the promising nanomaterial sensing in the nearest future.
